# Antimicrobial peptides of buffalo and their role in host defenses

**DOI:** 10.14202/vetworld.2018.192-200

**Published:** 2018-02-15

**Authors:** Khangembam Victoria Chanu, Dimpal Thakuria, Satish Kumar

**Affiliations:** 1 ICAR-Directorate of Coldwater Fisheries Research, Bhimtal - 263 136, Uttarakhand, India; 2 ICAR-Indian Veterinary Research Institute, Bareilly - 243 122, Uttar Pradesh, India

**Keywords:** antimicrobial peptides, *Bubalus bubalis*, cathelicidins, defensin, hepcidin

## Abstract

Antimicrobial peptides (AMPs) are highly conserved components of the innate immune system found among all classes of life. Buffalo (*Bubalus bubalis*), an important livestock for milk and meat production, is known to have a better resistance to many diseases as compared to cattle. They are found to express many AMPs such as defensins, cathelicidins, and hepcidin which play an important role in neutralizing the invading pathogens. Buffalo AMPs exhibit broad-spectrum antimicrobial activity against both Gram-positive and Gram-negative bacteria. Similar to its natural form, synthetic analogs of buffalo AMPs are also antimicrobial against bacteria and even fungus making them a good target for the development of therapeutic antimicrobials. In addition to its antimicrobial effect, AMPs have been demonstrated to have a number of immunomodulatory functions, and their genes are responsive to infections. Further, induction of their gene expression by external factors may help in preventing infectious diseases. This review briefly discusses the AMPs of buffalo identified to date and their possible role in innate immunity.

## Introduction

Domestic water buffalo (*Bubalus bubalis*) is a major milk-producing animal in several countries and a significant contributor in global milk production (http://www.fao.org). India is the largest milk producing country, and as per the FAO 2009 statistics, 56.1% of total milk production in India is contributed by buffalo. India is also world’s largest exporter of buffalo meat, which forms about 30% of total meat production in the country (http://ficci.in). According to the 19^th^ livestock census, 2012, the total number of buffalo in the country is 108.7 million showing a growth of 3.19% since 2007 (http://dahd.nic.in). It showed that these animals are an important section of livestock wealth of the country. Water buffaloes are generally healthy animal considering the hot, humid environment where they live. Although these regions are favorable to infections, there is a less deleterious effect of diseases on buffalo as compared to that of cattle in the same ecosystem (http://www.buffalopedia.cirb.res.in). It indicates that buffaloes have a stronger innate immunity to fight against infection. It was also reported that buffalo polymorphonuclear cells contain an array of antimicrobial proteins and peptides which play a significant role in the immune defensive system [1]. Antimicrobial peptides (AMPs) are evolutionary conserved essential molecules of the innate immune system which are found among all classes of life. For example, bacteria produce bacteriocins to inhibit the growth of similar or closely related bacterial strain [2]. Certain fungi of *Trichoderma* genus produce peptaibols, peptides containing α-aminoisobutyric acid, and ending in an alcohol [3]. Cecropins, first isolated from the hemolymph of *Hyalophora cecropia*, constitute the main part of cell-free immunity in insects [4]. Fish expresses a fish-specific class of the cecropin family, called piscidins [5]. Magainins and dermaseptins are AMPs isolated from frogs [6,7]. In avian species, cathelicidins and β-defensins are two major families of AMPs [8]. Cathelicidins and defensins also constitute the main mammalian AMPs [9].

In animals, AMPs are believed to be the first line of defense and are mostly found in the tissues and organs that are exposed to airborne pathogens [9,10]. AMPs have been demonstrated to kill bacteria, fungus, enveloped viruses, and even cancer cells [11-16]. In recent years, AMPs have gained the interest of many researchers because of its broad-spectrum activity against several pathogens and have been studied in different species. In buffalo, AMPs studied so far belong to defensins, cathelicidins, and hepcidin and new members are being discovered. Considering the importance of buffalo in livestock sector and beneficial effect of these naturally occurring molecules, this review provides an overview of AMPs identified in *B. bubalis* with their role in immunity.

## Defensins

Defensins comprise an important family of AMPs. They are small (29-45 amino acid residues) cationic peptides that contain six conserved cysteine residues, which form three disulfide bonds [17]. Defensins are classified into alpha-, beta-, and ­theta-defensins based on size and pattern of disulfide bonding. Alpha-defensins have been identified in humans, monkeys, and several rodent species. Theta-defensins are the only cyclic peptides of animal origin and are believed to have evolved from alpha-defensins [18]. They have been isolated from rhesus monkeys and baboons but not from humans, chimpanzees, and gorillas [19,20]. Beta-defensins have been found in every mammalian species explored so far and are involved in protecting the skin and the mucous membranes of the respiratory, genitourinary, and gastrointestinal tracts [20]. Beta-defensins are encoded by genes consisting of two exons with the first exon encoding the signal sequence and the second encoding the propeptide and mature peptide [21]. They are synthesized as pre-propeptides and are post-translationally processed into mature, active peptides.

**Figure-1:**
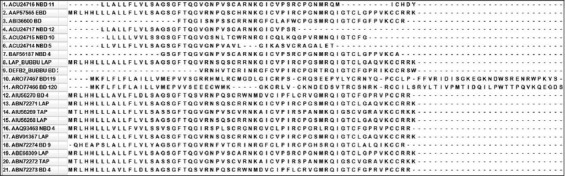
Alignment of beta defensin sequences of buffalo. Primary amino acid sequences with accession number were obtained from NCBI and aligned by ClustalW of MEGA 7.0 [97].

Alignment of primary amino acid sequences of defensins precursor sequences from buffalo shows that lingual AMP (LAP), tracheal AMP (TAP), enteric beta-defensin (EBD), and beta-defensin 4 (BD4) have high sequence identity (Figure-1). There was 100% identity in the sequences of LAP isolated from mastitic udder (ABN72271), teat canal (AIU56268), and female reproductive tract (ABV01367) of buffalo. However, LAP from the tongue (ABE66309) showed only 87.5% identity with LAP from mastitic udder, teat canal, and female reproductive tract. EBD from distal ileum (AAP57565) exhibited 93.8%, 92.2%, 92.2%, and 92.2%, respectively, with LAP from tongue, mastitic udder, teat canal, and female reproductive tract. There was 100% identity in the sequences of BD4 from mastitic udder (ABN72273) and BD4 from teat canal (AIU56270). Neutrophil BD4 had an identity of 71.4% with LAP of mastitic udder, teat canal, and female reproductive tract. BD 120 (ARO77466) and BD119 (ARO77467) isolated from the uterus of Egyptian buffalo showed sequence identity lesser than 25% with other defensins in the alignment. TAP from teat canal (AIU56269) and TAP from mastitic udder (ABN72272) showed 100% sequence identity. TAP from bovine was the first well-characterized member of the beta-defensin family [22]. It is a 38 amino acid peptide and the putative precursor was predicted to be 64 amino acids. The purified peptide showed broad-spectrum antimicrobial activity against *Escherichia coli*, *Staphylococcus aureus*, *Klebsiella pneumonia*, *Pseudomonas aeruginosa*, and *Candida albicans*. In buffalo, cationic peptides from tracheal epithelial cells have been isolated and characterized [23]. The peptides were smaller than 10 kDa and showed antibacterial activity against *E. coli* and *Salmonella typhimurium*. The antimicrobial activity of TAP is thought to result from disruption of bacterial membranes and pore formation which results from the electrostatic interaction of the positively charged peptide and negatively charged phospholipid of the bacterial membrane [24,25]. In tracheal tissues of buffalo, beta-defensin 4, beta-defensin 10, and beta-defensin 11 have also been identified [26]. Other than respiratory tract, TAP expression has also been found in teat canal mucosal epithelium and mastitic udder of buffalo [27,28]. This indicates that mucosal epithelial lining in buffalo is gifted with cationic AMPs to fight against microbial invasion.

**Figure-2:**
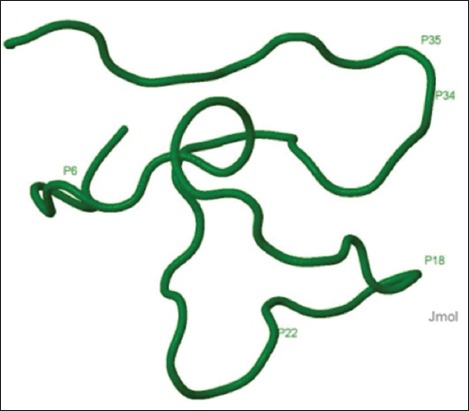
Predicted three-dimensional structure of buffalo lingual antimicrobial peptide (Accession number: ABE66309). The structure was predicted by PEP-FOLD and visualized in Jmol [98,99]. Proline residues are labeled on the molecule.

LAP was first isolated from the inflamed tongue of cattle but found more extensively throughout the body and detected even in milk [29-31]. Expression of LAP selectively increases in inflamed areas showing a closer relationship with immune response than simple antimicrobial activity. In buffalo, LAP has been identified from different tissues [32-37]. Precursor protein of buffalo LAP is of 64 amino acids and the matured AMP is of 42 amino acids [35]. It has been suggested that mature active peptides are released quickly from precursor by proteolytic activity during microbial invasion [38,39]. The mature peptide has the characteristic features of beta-defensin family including six conserved cysteine residues. In addition to its eight positively charged and eight hydrophobic amino acids in the mature peptide of buffalo LAP, the presence of proline residues might be responsible for making it potent endogenous AMP (Figure-2). It has been documented that proline-rich peptide can enter the cell without membrane lysis, bind to the ribosome, and interfere with the process of protein synthesis [40,41]. Proline also enhances the microbicidal activity by forming flexible helical link which increases membrane permeability and allows the hydrophobic residues to reside in the concave helical region [42,43]. Synthetic analogs of buffalo LAP showed antimicrobial activity against *S. aureus*, *Listeria monocytogens*, *E. coli*, and *S. typhimurium* [44]. Beta-defensin peptides, namely, LAP and BNBD-2 (Bovine neutrophil beta-defensin-2) isolated from mastitic milk of buffalo also showed significant antibacterial activity against *S. aureus* and *E. coli* [45]. The expression of these AMPs in the ductal epithelium of mammary gland might has been induced during mastitis leading to release of these peptides in the milk. As observed in the alignment of sequences, LAP identified in tissues other than the tongue of buffalo has reduced number of proline residues [32,33,35-37]; hence, a comparative study may be carried out to understand the role of proline in these AMPs. Another neutrophil beta-defensin, BNBD-4, was identified earlier from bone marrows of water buffalo [46]. From distal ileum of buffalo, EBD mRNA was characterized which encodes for a 64 amino acids precursor peptide [47]. EBD was first identified in the intestine of cattle and expressed highly in distal small intestine and colon, and its expression was inducible by gastrointestinal infection with *Cryptosporidium parvum* suggesting the participation of these molecules in local host defense of enteric mucosa [48]. In the mature peptide of buffalo EBD, the amino acids, serine (S), leucine (L), and tryptophan (W) at positions, 26, 33, and 63, respectively, of cattle EBD were found to be replaced by positively charged basic amino acid arginine (R). These substitutions increase the net positive charge of buffalo EBD and perhaps enhance the antimicrobial activity which may be correlated with better disease resistance in buffalo [47].

## Cathelicidins

Cathelicidins are proteolytically activated small, cationic AMPs with broad-spectrum antimicrobial activity against bacteria, enveloped viruses, and fungi [49]. The name cathelicidin was given to this group of AMPs because its prosequences showed high sequence identity with the sequence of a protein termed cathelin, which is isolated from porcine leukocytes [50,51]. Cathelicidin precursors comprise in most cases, a highly identical N-terminal pre-prosequences and a variable C-terminal antimicrobial domain. Matured cathelicidins are released by elastase enzyme by cleavage at a specific valine residue of the precursor [50]. In general, cathelicidins exhibit its antimicrobial activity by disintegrating the cell membrane of organisms [49]. Certain cathelicidins such as porcine cathelicidins PR-39 and indolicidin, a member of cathelicidin, were reported to inhibit protein synthesis and even induce the degradation of certain necessary proteins for DNA replication by the pathogen [52]. Indolicidin had been shown to bind DNA at preferred sequence, which may contribute to its antimicrobial action and can also inhibit topoisomerase 1, enzymes that cut one strand of double-stranded DNA, relax the strand, and reanneal the strand [53,54].

**Figure-3:**
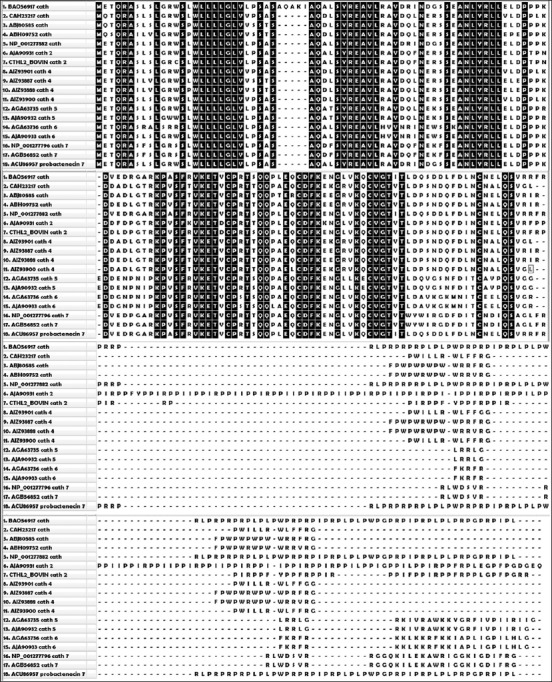
Alignment of buffalo cathelicidin by ClustalW of MEGA 7.0 [97]. The sequences with accession number were obtained from NCBI. Partial sequences were not included in the alignment. 100% conserved sites are toggled.

**Figure-4:**
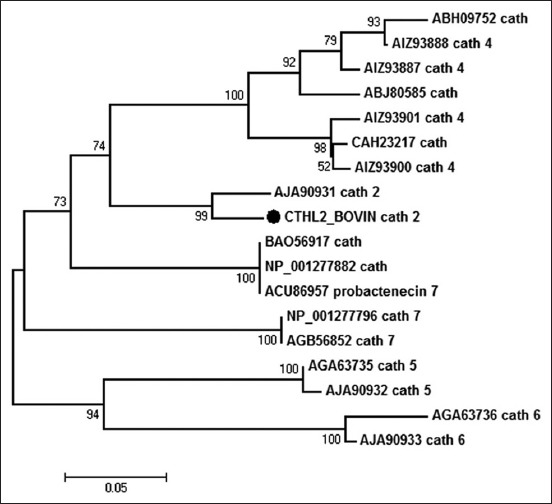
Phylogenetic relationships of different buffalo cathelicidins. Presented is a bootstrapped (1000 trials) neighbor-joining phylogenetic tree showing the evolutionary relationship of buffalo cathelicidin protein sequences [100,101]. Cattle cathelicidin 2 (CTHL2_BOVIN) was also used for comparison with buffalo cathelicidin 2.

Human and mice have only one cathelicidin gene while other mammals such as cow, sheep, pig, and rabbit can have many [55]. The first mammalian cathelicidins were bactenecins (Bac-5 and Bac-7) isolated from bovine neutrophil [56]. Both Bac-5 and Bac-7 efficiently kill *E. coli, S. typhimurium*, and *K. pneumonia* and arrest the growth of *Enterobacter cloacae, S. aureus*, and *Streptococcus agalactiae*. In buffalo, a cathelin-like 10.5 kDa region of 91 residues was identified from myeloid cells [57]. Buffalo uterus also expresses cathelicidin which may prove to be a potent antimicrobial agent [58]. A number of cathelicidins have been identified in buffalo and the alignment of the sequences showed high variability at C-terminal antimicrobial domain (Figure-3). When cathelicidin 2 of buffalo (AJO90931) was compared with bovine cathelicidin 2 (CTHL2_BOVIN), a sequence diversity 8.4% was observed. Within the buffalo cathelicidins, probactenecin 7 from bone marrow (ACU86957) showed 33.5%, 28.5%, and 28.4% sequence divergence to cathelicidins obtained from male reproductive organ (ABH09752), female reproductive tract (ABJ80585), and bone marrow (CAH23217), respectively. All the cathelicidins 4 (AIZ93887, AIZ93888, AIZ93900, and AIZ93901) used in the alignment exhibited sequence divergence from each other ranging from 2.1 to 15.4%. Cathelicidin 5 (AGA63735 and AJA90932) also showed a sequence diversity of 1.3%. There was a 3.9% diversity between the sequences of cathelicidin 6 (AJA90933 and AGA63736). There was no divergence in the sequence between cathelicidin 7 like precursor (NP_001277796) and cathelicidin 7 (AGB56852). This diversity may be due to pathogen-driven selection endowing superior antimicrobial activity which is a possible explanation of the role of cathelicidins in innate immunity of buffalo [59]. The percent variation among buffalo cathelicidins is also presented in the form of a phylogenic tree (Figure-4) which also represents the evolutionary lineage of cathelicidins.

The first cathelicidin reported in buffalo was myeloid cathelicidin which is homologous to *Bos taurus* cathelicidin-4 also known as indolicidin [60]. Soon after, another cathelicidin which appeared to be homologue of *B. taurus* cathelicidin-7 was identified [61]. Buffalo cathelicidin 4 shows high polymorphism and their structurally diverse homologs were also identified [59]. AMPs of these newly identified cathelicidin 4 subtypes produced antimicrobial effect by disrupting the membrane integrity of bacteria and caused blebbing, budding, and pore-like structure formation. Another three novel myeloid cathelicidins BuMAP-28, BuMAP-29, and BuMAP-34 having 28, 29, and 34 residues, respectively, at the C-terminal putative antimicrobial domain were predicted from cDNA sequences [62]. Synthetic BuMAP-34 showed antibacterial activity against *Riemerella anatipestifer*, *K. pneumoniae*, *S. typhimurium*, *S. aureus*, and *C. albicans* [63]. Further, biocomputational analysis of buffalo cathelicidin 3 indicated that different types and transcript variants of cathelicidins within the same species might have resulted from positive selection during evolution in response to pressure exerted by the pathogens [64]. Some of the recent additions of buffalo cathelicidins at NCBI protein database include cathelicidin 1, 2, 3, 5, 6, and 7 and variants of cathelicidin 4 [65-73]. The presence of diverse cathelicidins in buffalo and their broad-spectrum antimicrobial activity indicates that these peptides play an important role in neutralizing invading pathogens.

## Hepcidin

**Figure-5:**
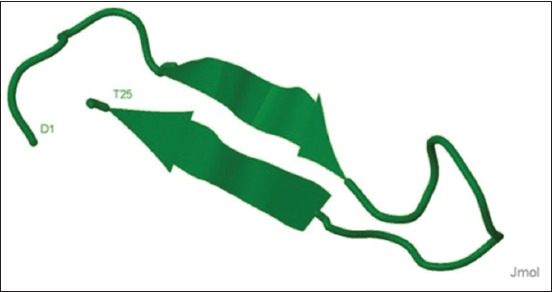
Predicted three-dimensional structure of buffalo hepcidin. The structure was predicted by PEPFOLD and visualized in Jmol [98,99].

**Figure-6:**
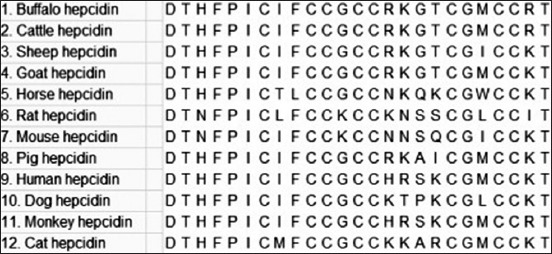
Matured peptide sequences of hepcidin. Precursor protein sequences were derived from NCBI, trimmed to 25-mer matured peptide sequences, and aligned by ClustalW of MEGA 7.0 [97]. All the sequences showed conserved 8 cysteine residues. Goat and cat hepcidin are predicted sequences.

Hepcidin is a 25-residue, cysteine-rich cationic AMP. It is synthesized as a precursor protein which undergoes two enzymatic cleavages to release the biologically active 25-mer peptide [74,75]. There are eight cysteine residues in mature peptide which form intramolecular disulfide bonds [76]. Three forms of hepcidin with N-terminal truncations, with 20, 22, and 25 residues, have been characterized from human urine and all the forms were with 8 cysteine residues. Human hepcidins are active against *S. aureus, S. epidermidis, E. coli, C. albicans, Aspergillus fumigatus*, and *Aspergillus niger* [75]. Hepcidins have been reported from different species, and there are more than 2000 hepcidin sequences available in databases. The deduced amino acid sequence of matured buffalo hepcidin consisted of 25 amino acids with 8 cysteine residues [77] and formed a distorted beta-sheet structure with a hairpin loop (Figure-5). On alignment, matured buffalo hepcidin showed 100% amino acid sequence identity with cattle hepcidin and minimal differences with that of other mammalian species (listed in Figure-6) indicating its highly conserved nature across species. As observed in the alignment, of 25 amino acids, matured buffalo hepcidin was found to differ by 2, 1, 7, 9, 8, 3, 5, 6, 4, and 5 amino acids from that of sheep, goat, horse, rat, mouse, pig, human, dog, monkey, and cat, respectively. Synthetic analogs showed antibacterial activity against *S. aureus* with low cytotoxic effect on buffalo lymphocytes [78]. It was already reported that hepcidin at a concentration more than 3000-fold as found in human urine is not cytotoxic [75]. Similarly, reports on toxic concentrations of AMPs (new designed cationic AMPs and modified alpha-helical model peptide) to mammalian cells also stated that their toxicity is commonly higher by one log of magnitude than that of minimum inhibitory concentrations against bacteria [79,80]. Therefore, hepcidin may be targeted for the development of potent antimicrobial agent. In addition to its antimicrobial activity, hepcidin also acts as a negative regulator of iron. Hepcidin-mediated iron regulation decreases iron in blood which is thought to increase host resistance to microbial infection [81]. Overall, it indicates that hepcidin plays a crucial role in host immune defense against microbial infections.

## Immunomodulatory Effect and Therapeutic Potential of AMPs

Recently, AMPs are being referred as host defense peptides by many authors owing to the fact that these peptides act mainly through immunomodulation [12]. Immunomodulatory activities of these peptides include modulation of the production of pro-inflammatory and anti-inflammatory cytokines and chemokines, recruitment of immune cells, induction of cellular differentiation and activation, regulation of cellular processes such as autophagy, apoptosis, and pyroptosis, and also the promotion of wound healing [12,82,83]. For example, certain family members of beta-defensin have been revealed to chemoattract immature dendritic cells and CD45RO+ CD4+ T cells through chemokine receptor CCR6. Further, it was also demonstrated that human beta-defensin 2 and 3 and mouse beta-defensin 4 and 14 could also chemoattract macrophages and monocytes through CCR2, a chemokine receptor expressed on monocytes, macrophages, and neutrophils [84]. Beta-defensins activate primary macrophages and also enhance pro-inflammatory responses to support inflammatory reactions initiated by toll-like receptor (TLR) ligands [85]. Similarly, LL37 (human cathelicidin) is known to impact the macrophages to produce pro-inflammatory cytokines in response to TLR agonists [86]. In addition, LL37 was shown to produce its ­immunomodulatory effect through activation of receptors, FPR2 and P2X7, a receptor highly expressed in immune cells [87,88]. Due to its multifunctionality as broad-spectrum antimicrobial against bacteria, yeasts, fungi, and viruses in addition to its cytotoxicity against cancer cells, anti-inflammatory, and immunomodulatory activities, AMPs have garnered interest as novel therapeutic agents [89]. By introducing some selective changes to the naturally occurring peptides, analogs with increased potency against microorganisms but reduced toxicity toward mammalian cells have been developed [90]. For designing novel antibiotics using the native AMPs as templates, some of the strategies involved are to change from L- to D-amino acids, C-terminal amidation, and N-acetylation [91]. For example, C-amidation and N-acetylation of octapeptide (RGKAKCCK), derived from human beta-defensin 1, significantly improved its antimicrobial activity with no toxicity to human cell even at high concentration and therefore appropriate for therapeutic applications [92]. Several AMPs are currently being evaluated not only for its antimicrobial effect but also as new pharmacological agents to modulate the immune response, promote wound healing, and prevent post-surgical adhesions [93]. It had been demonstrated that application of hBD2 or hBD3 promotes the healing of *S. aureus*-contaminated bone defects [94]. Similarly, LL-37 is developed for the treatment of chronic leg ulcers, and its clinical trial showed that LL-37 has a significantly improved healing rate compared to placebo [95,96].

## Conclusion and Future Prospects

With increased interests on AMPs, the list of buffalo AMPs is ever expanding. Many of the studies have focussed on the characterization of cDNA and its deduced peptide sequence. However, isolation and analysis of antimicrobial activity of these peptides are very limited as compared to other species. There are only few reports on the antimicrobial activity of synthetic analogs of buffalo AMP. Moreover, experimental studies on the inducibility of the genes encoding these peptides in buffalo are also lacking. Therefore, functional studies of these peptides along with its expression analysis during encounter with pathogenic microbes will be helpful in better understanding of the immune defense system of buffalo. For therapeutic applications, synthetic analogs of buffalo AMPs may be developed following some strategies to modify the naturally occurring peptides to improve its efficacy and host cell toxicity. Incorporation of unnatural amino acids (D-amino acids) at suitable positions may be done to enhance resistance of AMPs to proteases and sustain their activity for longer duration. Modifications in the synthetic analogs to get shorter length as compared to native sequences but with improved activity may also help to reduce the costs of production. Further, AMPs can also be synthesized in dendrimeric form to enhance its antimicrobial effect. The immunomodulatory effect of these AMPs (or synthetic analogs) on host body may also be investigated by evaluating the induction of chemokines, anti-inflammatory properties, and activation of signaling pathways.

## Authors’ Contributions

All the authors conceptualized the manuscript. Both KVC and DT drafted the manuscript. SK has given the intellectual suggestions. KVC has done the software analysis part. DT and SK critically reviewed the manuscript. All the authors have read and approved the final version of the manuscript.
